# An intraosseous malignant peripheral nerve sheath tumor of the lumbar spine without neurofibromatosis: Case report and review of the literature

**DOI:** 10.3892/ol.2014.1987

**Published:** 2014-03-21

**Authors:** KAYO SUZUKI, TAKETOSHI YASUDA, TAKESHI HORI, KENTA WATANABE, MASAHIKO KANAMORI, TOMOATSU KIMURA

**Affiliations:** 1 Department of Orthopaedic Surgery, Faculty of Medicine, University of Toyama, Toyama City, Toyoma 939-0194, Japan; 2 Department of Orthopaedic Surgery, Iiyama Red Cross Hospital, Iiyama City, Nagano 389-2295, Japan; 3 First Department of Human Science, Faculty of Medicine, University of Toyama, Toyama City, Toyoma 939-0194, Japan

**Keywords:** intraosseous malignant peripheral nerve sheath tumor, spine, total *en bloc* spondylectomy

## Abstract

A malignant peripheral nerve sheath tumor (MPNST) is defined as any malignant tumor that develops or differentiates from cells in the peripheral nerve sheath. This tumor is commonly associated with neurofibromatosis type 1 (NF1) and previous radiotherapy treatment. Primary intraosseous MPNSTs are extremely rare and a case of the lumbar spine in a patient without NF1 is reported in the present study, with a review of the intraosseous MPNST literature. A 45-year-old female presented with a 1-month history of severe lower back pain and pain radiating to the left leg. A total *en bloc* spondylectomy of L4 was performed. The postoperative histopathological diagnosis was MPNST with deletion of *NF1*, confirmed by dual-color fluorescence *in situ* hybridization (FISH) analysis. The tumor recurred 1 month following the surgery. Although adjuvant chemotherapy was administered, the patient succumbed due to intramedullary dissemination and carcinomatous meningitis 8 months following the initial consultation. *NF1* deletion by FISH analysis may be particularly useful in distinguishing MPNST from other high-grade malignancies with overlapping morphological features.

## Introduction

A malignant peripheral nerve sheath tumor (MPNST) is a rare tumor that accounts for ≤5% of soft-tissue sarcomas, often arising from Schwann cells (1). Approximately 50% of MPNSTs occur in patients with neurofibromatosis type 1 (NF1), and 10% of these are radiation-induced, while the remainder affect individuals without a known genetic predisposition. The most common locations for MPNSTs are the trunk, extremities and head and neck (2). Isolated studies have reported the development of MPNST following irradiation and primary or metastatic intradural MPNST of the spine (3–5). Spinal MPNST usually develops from spinal nerve roots and causes secondary bony changes. However, primary intraosseous MPNST is extremely rare and, to the best of our knowledge, only five cases in the spine have been reported in the literature (6–10). As the tumors have a high malignancy and invasive natural course with a common recurrence and distant metastases, they have a poor prognosis, even with gross total resection.

An additional case of intraosseous MPNST of the lumbar spine in a patient without NF1 is presented in the current study, and the relevant literature on intraosseous MPNST is reviewed. The study was conducted following a clinical research review by the ethics committee of Toyama University Hospital (Toyama City, Japan). Informed consent was obtained from the patient, who was advised that the data from the case would be submitted for publication.

## Case report

### Case summary

**Figure 1:**
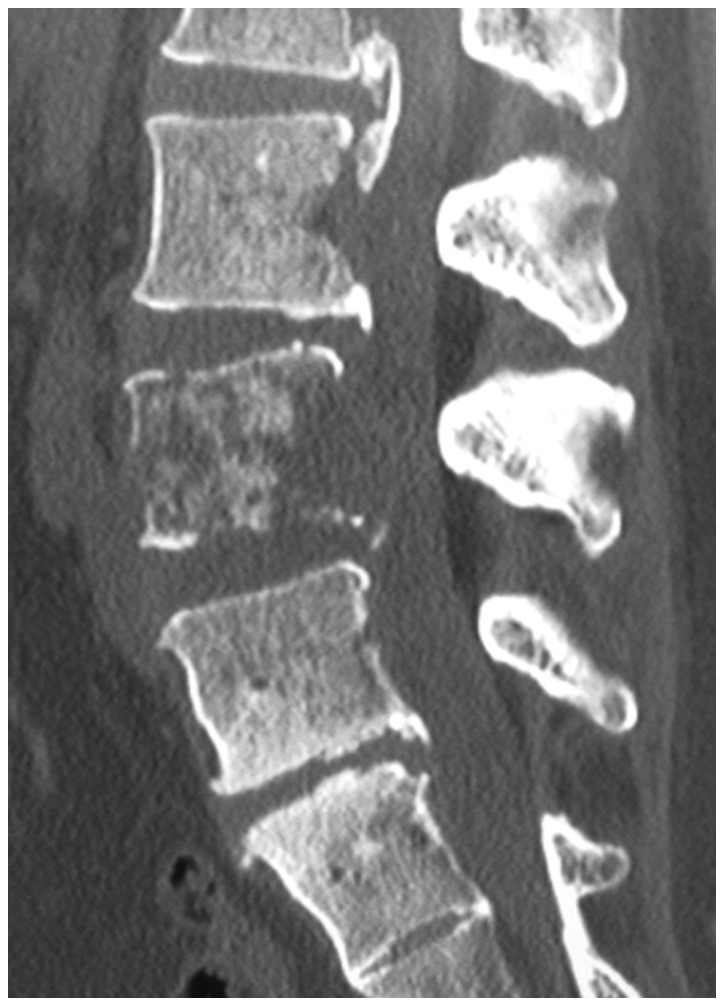
Computed tomography at the level of L4 reveals osteolytic destruction of the L4 vertebra with a defect on the posterior wall of the vertebra in the sagittal view.

**Figure 2:**
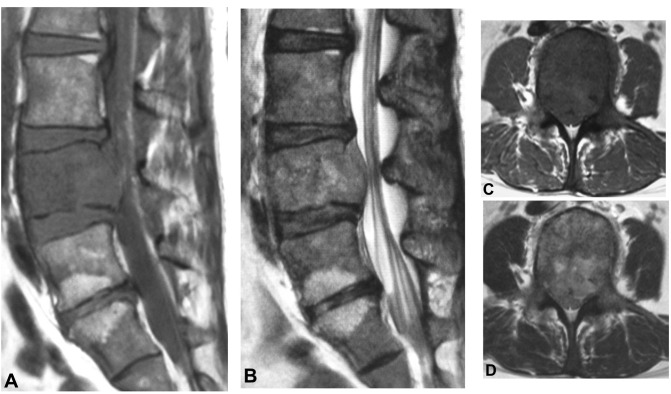
MRI of the lumbar spine. (A and C) T1- and (B and D) T2-weighted MRI of the lumbar spine in the (A and B) sagittal and (C and D) axial planes. The MRI shows L4 vertebral collapse and an extradural mass compressing the dura mater. The lesion extending from the L4 vertebral body to bilateral pedicles shows intermediate signal intensity on T1-weighted images and heterogeneous with mixed high and intermediate signal intensities on T2-weighted images. MRI, magnetic resonance imaging.

A 45-year-old female presented with a 1-month history of severe lower back pain and pain radiating to the left leg. No ‘café au lait’ spots or neurofibromas were present and the patient confirmed there was no family history of NF1. The neurological examination revealed a decreased sensation to a pin prick in the left L4 area. There was no obvious motor weakness of the leg, and bladder and bowel dysfunction was evident. Plain radiographs of the lumbar spine showed an osteolytic lesion at the L4 vertebral body. Computed tomography revealed compression of the spinal canal resulting from destruction of the posterior elements of L4 (Fig. 1). Magnetic resonance imaging (MRI) showed a destructive lesion and extradural tumor at the L4 level with dural compression. The lesion extending from the L4 vertebral body to bilateral pedicles showed intermediate signal intensity on T1-weighted images (Fig. 2A and C), and it was heterogeneous with mixed high and intermediate signal intensities on T2-weighted images (Fig. 2B and D). No other tumors were identified.

**Figure 3:**
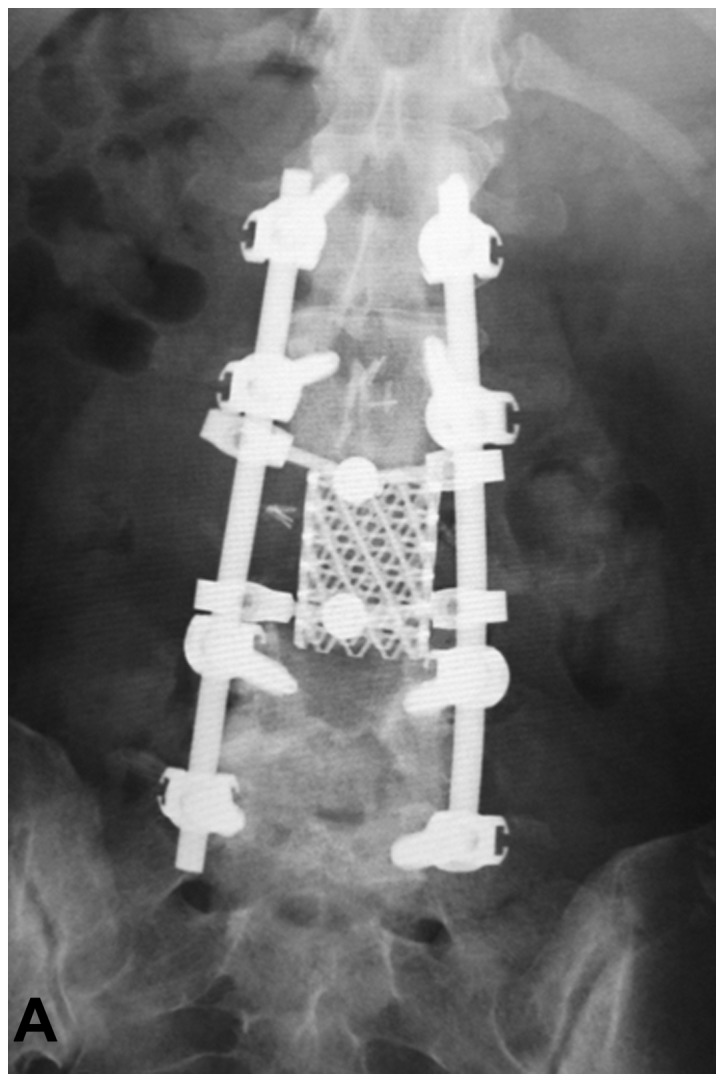

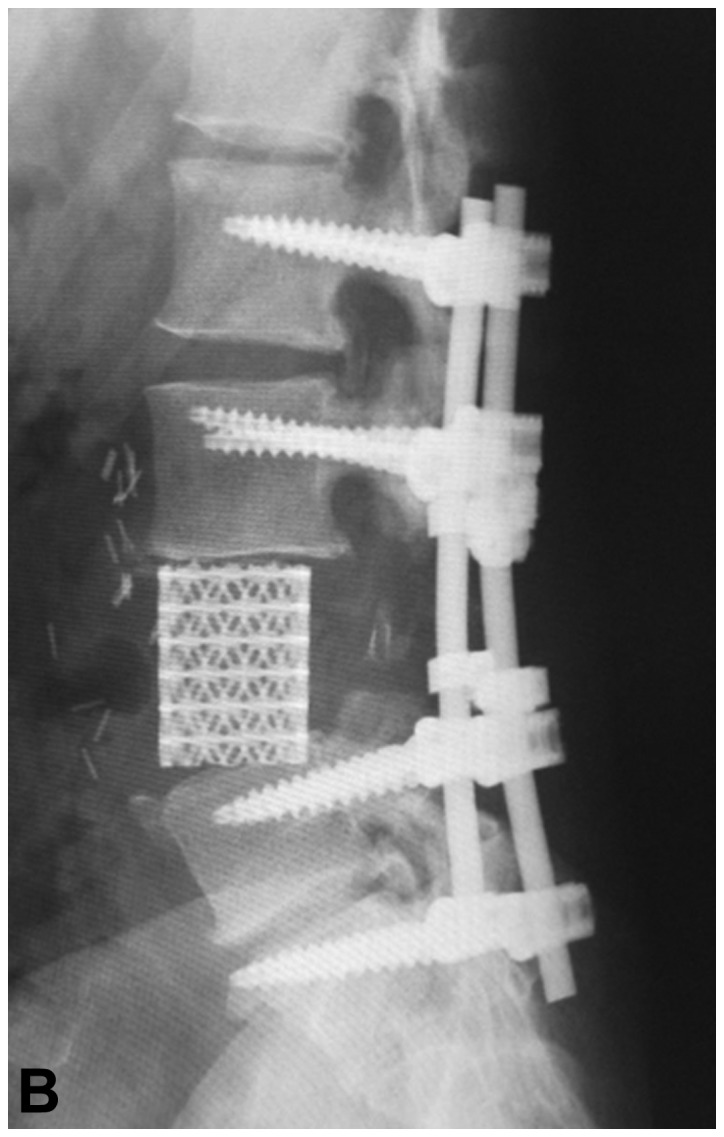
Radiographic findings following the second surgery. (A) Anterior-posterior and (B) lateral views.

As the paralysis progressed rapidly, decompressive laminectomies and extradural tumor resection were performed. At the same time, posterior spinal fusion with instruments including a percutaneous pedicle screw system (Mantis, Stryker Japan Co., Tokyo, Japan) was performed for maintenance of spinal stability and prevention of tumor dissemination. The tumor appeared slightly adherent to the dura. The tumor mass was not obviously connected with the bilateral L4 spinal nerve roots or the dura. The boundary between the tumor and the L4 vertebral body was unclear. Histologically, the tumor consisted of coagulation necrosis and sheets of tumor cells with alternating areas of hyper- and hypocellularity. The majority of the tumor cells were atypical spindle cells. The spindle cells exhibited mitotic figures and pleomorphism. Immunohistochemically, the spindle cells were positive for vimentin and smooth muscle actin and negative for S-100, epithelial membrane antigen (EMA) and cluster of differentiation 34 (CD34). The histological diagnosis was undifferentiated pleomorphic sarcoma. Postoperatively, the symptoms of the patient were dramatically relieved. However, they recurred two weeks following the surgery, and the patient presented with progressive hypoesthesia and motor weakness in the legs. A total *en bloc* spondylectomy was performed according to a previous study (Fig. 3) (11). Briefly, all posterior elements of the spine (the spinous process, the superior and inferior articular processes, the transverse process and the pedicle) were removed in one section using a posterior approach. Subsequently, the anterior elements of the spine (the vertebral body and psoas muscle) were removed *en bloc* at the coincident level of the posterior halves using an anterior midline transperitoneal approach. To maintain stability of the tumor resection, posterior and anterior instrumented fixation was performed.

Although multidrug adjuvant chemotherapy (30 mg/m^2^ doxorubicin, 1.5 g/m^2^ ifosfamide and 300 mg/m^2^ dacarbazine; 3 days] was administered following the second surgery (12), the patient succumbed to intramedullary dissemination and carcinomatous meningitis 8 months following the initial consultation.

### Pathological study

**Figure 4:**
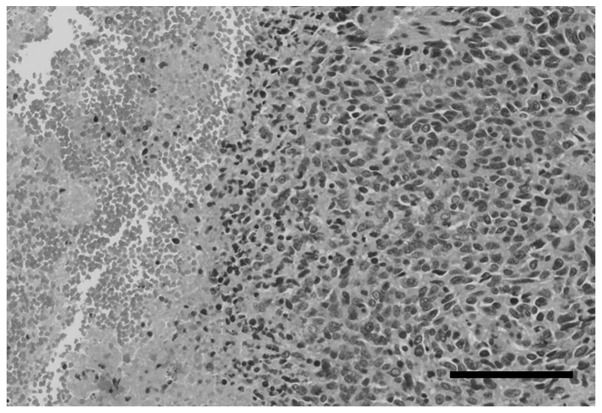
Histopathological findings of the specimen from the second surgery. The tumor consists exclusively of dense fascicles of spindled cells alternating with the area of coagulation necrosis. The spindle cells have irregular contours with abundant gross cellular atypia, mitotic figures and pleomorphism (hematoxylin and eosin stain; scale bar, 100 μm).

The second postoperative histopathological examination revealed a densely cellular area composed of spindle cells with a fascicular growth pattern and coagulation necrosis. Each of the spindle cells exhibited irregular contours with abundant gross cellular atypia, mitotic figures and pleomorphism (Fig. 4). On immunohistochemical staining, the tumor cells were positive for vimentin and bcl-2, focally positive for EMA, and negative for AE1/AE3, desmin, S-100, CD99 and CD34.

### Fluorescence in situ hybridization (FISH) analysis

**Figure 5:**
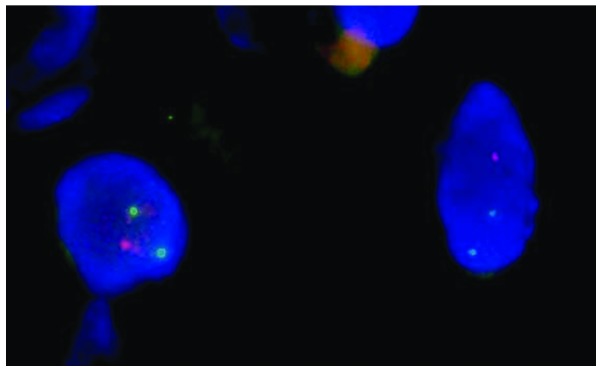
Dual color fluorescence *in situ* hybridization analysis showing the 17q deletion with two chromosome 17 MPO signals and one NF1 signal per nucleus (green, MPO region on 17q22; red, NF1 region on 17q11). MPO, myeloperoxidase; NF1, neurofibromatosis type 1. Magnification, ×100.

FISH was performed on an unstained, paraffin-embedded tissue section using the Dual Color, Break Apart Rearrangement Probe (Kreatech Diagnostics, Amsterdam, Netherlands) according to the manufacturer’s instructions. Hybridization signals were assessed in 200 interphase nuclei with strong, well-delineated signals and distinct nuclear borders by two individuals, as previously described (13). Dual color FISH analysis involving chromosome 17q showed that >10% of the cells from the tumor showed the deletion signal pattern of one red (*NF1* region probe on 17q11) and two green [myeloperoxidase (*MPO*) gene region on 17q22 as the control probe), demonstrating a deletion of the *NF1* gene (Fig. 5). These histopathological and cytomolecular findings confirmed the diagnosis of MPNST with focal epithelioid features.

## Discussion

MPNSTs are rare malignant tumors arising from peripheral nerve sheath cells. These tumors typically present as an enlarging mass originating from a peripheral nerve root of the trunk (~50%), in the extremities (~30%), or the head and neck region (~20%) (2). Secondary bony infiltration in a paraspinal MPNST is a well-known entity. However, a primary intraosseous MPNST is extremely rare. An intraosseous MPNST may develop from minute, mainly unmyelinated nerve roots that accompany nutrient vessels and ramify within Volkmann’s canals and bone marrow (14–16). To the best of our knowledge, only 19 cases of intraosseous MPNSTs, including the present study, have been reported in the literature to date (Table I) (6–10,17–29). There were nine male and 10 female patients, and the age at diagnosis ranged from 4–76 years. A total of eight of the 19 cases (42%) occurred in the mandible or maxilla and six cases (32%) involved a vertebral body of the spine. Although <50% of MPNSTs arise in patients with NF1 (29), intraosseous MPNST cases were not associated with the hereditary NF1 syndrome, except for one case. The case of the present study was also not associated with NF1.

Due to a wide morphologic spectrum and lack of specific markers, the diagnosis of intraosseous MPNST is extremely difficult, particularly in a case lacking the manifestations of NF1 and classic histopathological features. High-grade MPNSTs may resemble other malignancies, including fibrosarcoma, synovial sarcoma and malignant fibrous histiocytoma. In the present study, although the initial histological diagnosis was an undifferentiated pleomorphic sarcoma, the histological diagnosis of the recurrent mass was MPNST with positive immunohistochemistry for vimentin and bcl-2, focally positive for EMA, but negative for AE1/AE3, desmin, S-100, CD99 and CD34. Numerous antigens are useful in the identification of nerve sheath differentiation, including S-100 protein, Leu-7 and myelin basic protein. The protein S-100 is the most commonly used antigen for neural differentiation, and it can be identified in ~50% of MPNSTs, although the staining is typically focal and limited to a small number of cells. As encountering an MPNST with a strong and diffuse immunoreactivity for the S-100 protein is uncommon, such a staining pattern always indicates that other benign diagnoses should be reconsidered, particularly cellular schwannoma. The proteins myelin basic protein and Leu-7 have been identified in ~40 and ~50% of MPNSTs, respectively (30).

The molecular pathogenesis of MPNST remains poorly understood. In contrast to numerous other sarcomas, there is no pathognomonic chromosomal translocation and conventional cytogenetic studies typically yield complex, often near-triploid karyotypes without specific aberrations (1). However, loss involving chromosome 17q, the site of the *NF1*, has been detected in 25–50% of reported sporadic and NF1-associated cases, often in the form of chromosomal monosomy. Similarly, *NF1* deletion in FISH analysis may aid in distinguishing MPNSTs from other high-grade malignancies with overlapping morphological features. Perry *et al* (31) demonstrated that *NF1* deletions were detectable in 76% of sporadic and NF1-associated MPNSTs by FISH analysis and observed in five out of six high-grade MPNSTs that lacked S-100 protein immunoreactivity, which is considered to be a significant marker for Schwann cells. Additionally, it has been reported that of eight cases with MPNST, *NF1* deletion was detected within the S-100-positive cellular populations of four MPNSTs (50%), while S-100-negative nuclei were observed in all eight MPNSTs. These results indicated the prevalence of *NF1* deletion in MPNSTs, regardless of S-100 protein expression (32). In the case of the present study, immunohistochemistry for S-100 protein was negative in tumor cells, and *NF1* deletion by FISH was simultaneously detected. Therefore, MPNST in the present case was believed to be a high-grade malignancy from the viewpoint of the S-100 protein-negative cells, which represent dedifferentiated Schwann cells.

Surgical resection is the treatment of choice for MPNSTs. Complete tumor resection with negative margins remains the most effective treatment. A study by Wong *et al* (33) reported that the rate of *en bloc* resection in 128 patients with MPNSTs was 83% and, of these patients, only 48% had negative surgical margins. However, *en bloc* resection is often extremely complicated in cases of spinal MPNST due to the surrounding spinal cord, dura mater and large blood vessels. In previous studies, four out of the five vertebral MPNSTs underwent subtotal resection, and the outcomes of three cases included two who succumbed to disease and one who remained with disease (Table I). Adjuvant radiation therapy to a dose of >60 Gy improves local control of this disease (34). The effectiveness of chemotherapy for MPNST remains controversial. Certain success has been reported with doxorubicin use alone or in combination with other drugs (35). However, the effects of chemotherapy and radiotherapy are unclear.

In conclusion, the current study presents a case of intraosseous MPNST arising in a lumbar vertebra. Although *en bloc* resection of the tumor and adjuvant chemotherapy were performed, the patient succumbed to carcinomatous meningitis. Since the outcome remains poor, further studies on genetic therapy of the tumor based on its molecular pathogenesis are required.

## Figures and Tables

**Table I:** Summary of reported cases of intraosseous MPNST.

Case (ref.)	Age (years)/Gender	Location	NF1	Surgery	Adjuvant therapy	Outcome (months)
1 (17)	55/M	Ulna	-	CR	None	NED (20)
2 (18)	65/F	Mandible	-	NA	NA	NA
3 (19)	65/M	Mandible	-	Resection	RT	AWD (NA): recurrence
4 (20)	4/F	Mandible	NA	NA	NA	NA
5 (21)	28/M	Ulna	-	NA	NA	NA
6 (22)	11/F	Mandible	-	Resection	None	NED (6)
7 (23)	76/F	Mandible	-	NA	NA	NA
8 (24)	61/F	Maxilla	-	NA	NA	NA
9 (25)	47/F	Maxilla	-	Resection	RT, CT	DOD (22)
10 (26)	50/M	Mandible	-	NA	RT	DOD (12)
11 (27)	28/M	Femur	-	Resection	None	DOD (1): pulmonary metastasis
12 (28)	26/M	Femur	-	Resection	CT	DOD (15): pulmonary metastasis
13 (29)	29/M	Ulna	-	Resection	None	DOD (36): pulmonary metastasis
14 (6)	40/F	C2	NA	STR, PSF	None	DOD (12): pulmonary metastasis
15 (7)	59/F	T3	-	STR, PSF	RT, CT	AWD (46): bone metastasis
16 (8)	75/F	T7	-	STR, PSF	RT	DOD (6): pulmonary metastasis
17 (9)	41/M	C7	-	CR, PSF	CT	NED (24)
18 (10)	75/M	L3	-	STR	NA	NA
19 (present)	45/F	L4	-	STR, PSF	CT	DOD (8): carcinomatous meningitis

MPNST, malignant peripheral nerve sheath tumor; NF1, neurofibromatosis type 1; M, male; F, female; C, cervical; T, thoracic; L, lumbar; CR, complete resection; NA, not available; STR, subtotal resection; PSF, posterior spinal fusion; RT, radiotherapy; CT, chemotherapy; NED, no evidence of disease; AWD, alive with disease; DOD, dead of disease.
